# Alternate-day modified fasting diet improves weight loss, subjective sleep quality and daytime dysfunction in women with obesity or overweight: a randomized, controlled trial

**DOI:** 10.3389/fnut.2023.1174293

**Published:** 2023-05-18

**Authors:** Saeedeh Hosseini Hooshiar, Akram Yazdani, Sadegh Jafarnejad

**Affiliations:** ^1^Research Center for Biochemistry and Nutrition in Metabolic Diseases, Kashan University of Medical Sciences, Kashan, Iran; ^2^Department of Biostatistics and Epidemiology, Kashan University of Medical Sciences, Kashan, Iran

**Keywords:** alternate-day modified fasting, intermittent fasting, calorie restriction, sleep quality, Pittsburgh

## Abstract

**Background:**

Both sleep time and quality can be associated with overweight or obesity. In obese people, visceral fat tissue develops, which results in an increment in the production of cytokines. The increased production of inflammatory cytokines can disturb the sleep/wake cycle. Therefore, weight loss by reducing fat tissue can improve sleep disorders. Intermittent fasting diets are popular and effective diets that can decrease body weight and improve anthropometric data and body composition. The present study aimed to evaluate the effect of Alternate-day Modified Fasting (ADMF) on sleep quality, body weight, and daytime sleepiness.

**Methods:**

Classification of 56 obese or overweight women, based on age and body mass index (BMI), was done using stratified randomization. Then individuals were assigned to the ADMF group (intervention) or Daily Calorie Restriction (CR) group (control) using the random numbers table for 8 weeks. We measured the Pittsburgh sleep quality Index (PSQI), weight, BMI, and the Epworth sleepiness scale (ESS) as primary outcomes and assessed subjective sleep quality (SSQ), sleep latency, sleep disturbances, habitual sleep efficiency, daytime dysfunction, and sleep duration as secondary outcomes at baseline and after the study.

**Results:**

Following an ADMF diet resulted in a greater decrease in weight (kg) [−5.23 (1.73) vs. −3.15 (0.88); *P* < 0.001] and BMI (kg/m^2^) [−2.05 (0.66) vs. −1.17 (0.34); *P* < 0.001] compared to CR. No significant differences were found in the changes of PSQI [−0.39 (1.43) vs. −0.45 (1.88); *P* = 0.73] and ESS [−0.22 (1.24) vs. −0.54 (1.67); *P* = 0.43] between two groups. Also, following the ADMF diet led to significant changes in SSQ [−0.69 (0.47) vs. −0.08 (0.40); *P* = <0.001], and daytime dysfunction [−0.65 (0.57) vs. 0.04 (0.75); *P*: 0.001] in compare with CR diet.

**Conclusion:**

These results suggested that an ADMF could be a beneficial diet for controlling body weight and BMI. The ADMF diet didn’t affect PSQI and ESS in women with overweight or obesity but significantly improved SSQ and daytime dysfunction.

**Clinical Trial Registration:**

The Iranian Registry of Clinical Trials (IRCT20220522054958N3), https://www.irct.ir/trial/64510.

## Background

Sleep disorders affect nearly one-third of adults. The association between sleep quality and food intake has been shown in studies (1). Both people with severe and moderate obesity are affected by low sleep quality (2, 3). The increment of visceral adipose tissue results in the release of inflammatory cytokines that may lead to a disturbance of the sleep-wake cycle (3). Current studies have shown a bidirectional relation between sleep and oxidative stress and inflammation. It has been shown that extremely long sleep duration and sleep disturbances could be related to increased levels of IL-6 and c-reactive protein, while insufficient sleep duration with IL-6 (4). Therefore, both duration and sleep quality can ameliorate with a decrease in weight (5). Gangwisch et al. showed that higher BMI is related to lower sleep duration (6). The first-line therapy for the reduction of weight in individuals with obesity or overweight is calorie restriction (7). Adherence to conventional diets for weight loss is low because of daily energy restriction (8). In recent years, the fasting diet has been proposed as an unconventional diet for losing weight [17]. In addition to losing body weight, it improves metabolic health (9). Among the different fasting methods that have been investigated, the ADMF diet is known to be an effective diet to lose weight. ADMF comprises intermittent periods of feasting and fasting, on alternate days. Some studies have shown a 3–7% decrease in weight under an ADMF diet during 8–12 weeks (10). Compared to CR, intermittent fasting diets have exhibited greater participant compliance over longer periods (11). What is not clear is whether calorie restriction or a fasting diet will further ameliorate body weight (7) and, after that, affect the quality of sleep. Several studies suggest that alternate-day fasting compared with CR could preserve muscle mass and reduce visceral adipose area (12). A systematic review reported that alternate-day fasting diets reduced body weight similar to CR (7, 13). Hutchison et al. have shown that alternate-day fasting leads to greater weight loss compared with CR (14). Recent evidence has reported that calorie restriction increases the quality of sleep (15–17), but studies on the effect of intermittent fasting diets on the quality of sleep are limited. Teong et al. showed that a significant change wasn’t found between CR and intermittent fasting diet on the sleep quality of women with overweight or obesity (18). Therefore, to achieve more definitive results in this field, the effect of a method of fasting “ADMF” on body weight, sleep quality, and daytime sleepiness was investigated in this trial.

## Methods

### Design of study

This study was a randomized, controlled, trial to investigate the effect of an ADMF diet and CR diet on body weight, daytime sleepiness, and sleep quality in overweight/obese women for 8 weeks. Individuals were recruited from several health centers located in Kashan, Iran by simple random sampling. Then participants were randomly assigned into groups control (CR) and intervention (ADMF). The study protocol was registered at the Iranian Registry of Clinical Trials (IRCT20220522054958N3) and was approved by the Ethics Committee of Kashan University of Medical Sciences (IR.KAUMS.MEDNT.REC.1401.046). All patients gave written consent to participate in the study. Inclusion criteria included women between 18 and 50 years old and 40 >BMI ≥25. Exclusion criteria included pregnancy, breastfeeding, having a chronic disease such as hypertension, diabetes, gastrointestinal disorders, and heart disease, losing 1–2 kg of weight in the past month, the habit of smoking, alcohol abuse, taking specific medication or following a specific diet, taking dietary supplements for weight loss, overnight shifts, having psychological and mental disorders.

In this study, 56 women were recruited by Simple Random Sampling from some health centers located in Kashan considering the inclusion and exclusion criteria. Eligible individuals were stratified based on age and BMI to make sure homogeneity of between-group. Individuals per stratum were placed into an ADMF group or CR group after baseline investigations. The allocation sequences were generated by using random numbers table by an independent statistician. The statistician was blinded throughout the entire trial.

### Diet protocol

**Figure 1:**
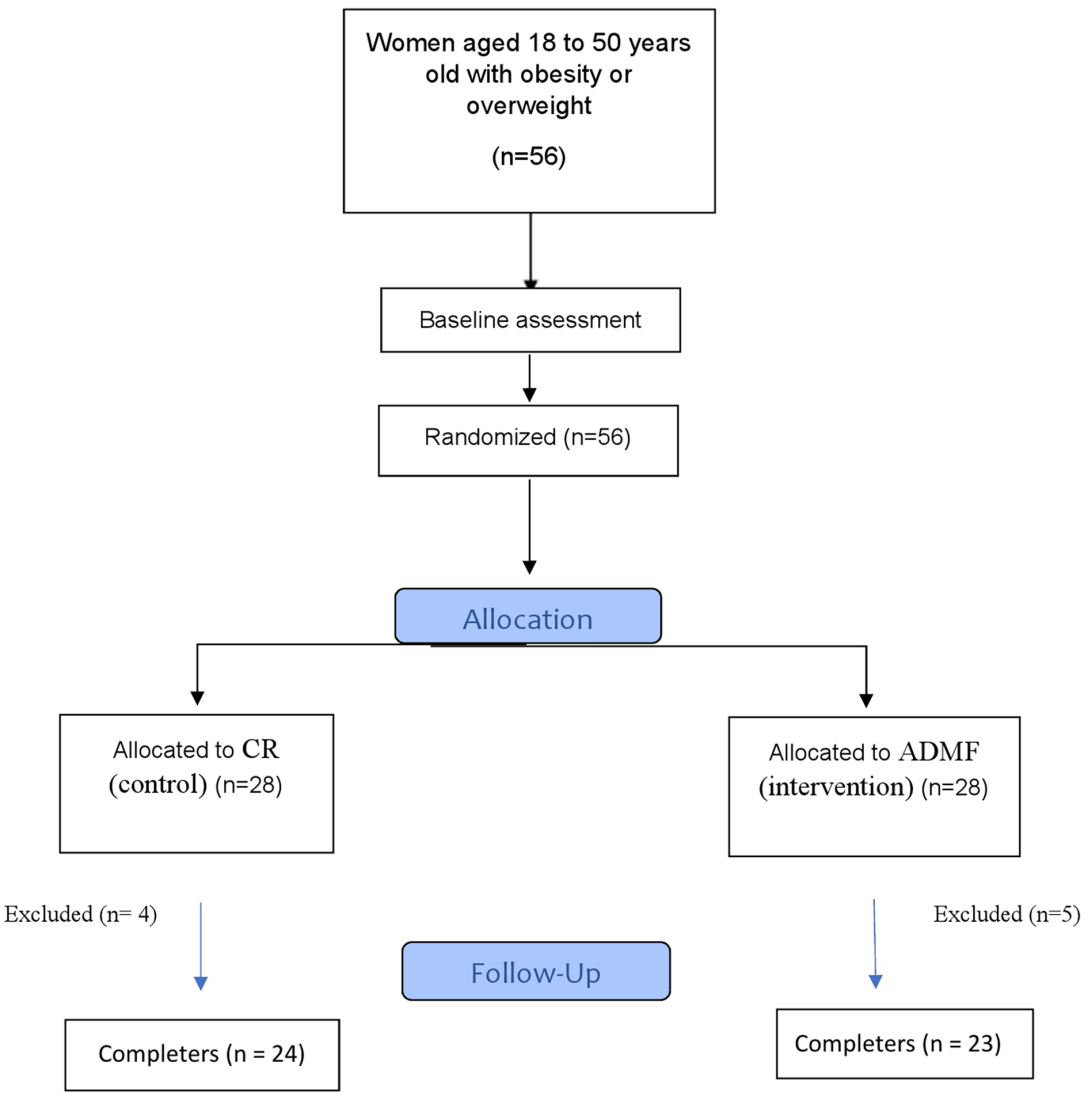
Flow chart of the study.

The flow diagram of the study has been presented in Figure 1. Individuals per stratum were placed into an ADMF group or CR group after baseline investigations. All individuals were needed to follow diets that were given to them based on daily energy requirements and their group for 8 weeks. The daily energy requirements of the participants were estimated by using the Mifflin equation (19). ADMF involved intermittent periods of fasting and feeding, every-other-day (on the fasting days, participants consumed only 25% of the daily recommended calorie and then on feeding days they consumed 100% of the estimated daily energy requirements). The fast and feed days started at midnight. All meals of the fast days were eaten as lunch between 12.00 p.m. to 2.00 p.m. to make sure that each participant was sustaining the same time of fasting. The consumption of non-starchy veggies (green leaf, cucumber, tomato, and lettuce) as well as energy-free beverages like water, tea, and coffee without sugar (less than 400 mg caffeine daily) was permitted. Participants were encouraged to drink plenty of water. The individuals consumed 100% of their daily energy needs (three main meals and three snacks) on feeding days and were asked to intake breakfast at 8:00 a.m., lunch at 13:00 and dinner at 8:00 p.m. Also, they were asked to eat their snacks at 10:00, 16:00, and 22:00. Participants of the CR group daily consumed 63% of their calculated calories (three main meals and three snacks) and were asked to take their main meals at 8:00, 13:00, and 20:00, respectively. Also, they were asked to take their snacks at 10:00, 16:00, and 22:00. The duration of the diet for both groups was 8 weeks. All participants in intervention and control groups needed to cook all their meals in their houses. Daily dietary protein, fat, and carbohydrates, accounted for 15, 30, and 55% of energy needs, respectively. Intervention and control group patients were required to keep their regular physical activity over the trial. All participants had the same number of calls to the dietician.

### Adherence to the diet

Participants’ adherence to the regimen was monitored every two weeks by completing food record questionnaires three times a week (two normal days and one day off) (20) The questionnaires were compared to the prescribed diet to assess adherence. To ensure accurate completion of the questionnaires, all participants received instructions on how to complete the forms, which included selecting appropriate days and units of measurement. The information obtained from the questionnaires was converted to grams using a home scale guide and analyzed with N4 software (First Databank Inc.; Hearst Corporation), which was adapted for Iranian foods. The software calculated the amount of energy and macronutrients received. Adequate adherence was defined as a total caloric intake between 80 and 110% of the prescribed amount (21). Participants whose daily calorie or macronutrient intake was less than 80% or more than 110% of the recommended amount were excluded from the study. Additionally, participants were contacted by phone every two weeks and monitored to answer any questions and encourage adherence to the diet and study protocol.

### Assessment of variables

The impact of ADMF and CR on primary outcomes (body weight, BMI, PSQI, and ESS) and secondary outcomes (SSQ, sleep latency, sleep duration, sleep efficiency, daytime dysfunctions, sleep disturbances) was assessed by change from baseline to end of the intervention.

### Weight and BMI

Participants’ weight, without shoes and with light clothes, was evaluated using the scale with an accuracy of 0.1 kg. A stadiometer with an accuracy of 0.5 cm was used to measure the height of patients. BMI was computed as weight (in kilograms) divided by height (in meters squared).

### Sleep quality

The sleep quality in the previous 4 weeks is evaluated self-reportedly by the PSQI. The PSQI questionnaire has 19 items and assesses 7 components of sleep: SSQ, sleep latency, sleep duration, use of sleeping medication, sleep disturbance, habitual sleep efficiency, and daytime dysfunction (22). The items are rated on a 4-point Likert scale in terms of the severity of the problem or frequency. The score range of each item is from 0 to 3. The total PSQI score has a range between 0 and 21. Higher scores express lower sleep quality (22).

### Daytime sleepiness

In this study, daytime sleepiness was measured by the ESS, a self-reported questionnaire. Participants answered items based on how likely they were to fall asleep or doze off during sedentary activities. ESS is an eight-item questionnaire and includes a respondent format “high chance of dozing” = 3, “moderate chance of dozing” = 2, “slight chance of dozing” = 1, and “would never doze” = 0. The total ESS score was calculated by summing the total scores of eight items. The total score range was from 0 to 24. Higher values indicate higher levels of sleepiness. The change scores were evaluated as the changes between the total ESS scores at the beginning and the end of the study (23).

### Physical activity record questionnaire

The physical activity questionnaire, based on metabolic equivalents (MET), was used to evaluate physical activity in this trial. It consisted of nine activity levels, ranging from rest and sleep with a metabolic equivalent of 0.9 to intense activity with a metabolic equivalent of ≥6 (24). Each level (A: 0.9 MET, such as sleep and rest; B: 1.0 MET, such as sitting quietly; C: 1.5 METs, such as working at a computer; D: 2.0 METs, such as standing or washing dishes; E: 3.0 METs, such as light cleaning; F: 4.0 METs, such as bicycling; G: 5.0 METs, such as gardening; H: 6.0 METs, such as aerobics; and I: >6 METs, such as running) was described with examples of activities of that particular MET level, and by a drawing. The physical activity scale was created so that the amount of time spent on each MET activity level (15, 30, or 45 min, and 1–10 h) on an average 24-h weekday could be recorded. The participants indicated the number of times per day they participated in each of the nine levels of activities, numbered 1–9. The Physical Activity score was evaluated by multiplying the MET level of the activity by the number of times per day and summing all the activity scores together (24).

### Statistical assessment

The Kolmogorov-Smirnov test was applied to investigate the normality of data distribution. A Chi-square test was used to compare qualitative data between the two groups. An independent *t*-test was used to evaluate between-group differences in quantitative data. To compare the mean of the quantitative data (within the group) at the beginning and end of the study, the paired *t*-test was used in parametric conditions and the Wilcoxon test was used in non-parametric conditions. To compare the mean between the two groups, a *t*-test was used in parametric conditions and the Mann-Whitney test was used in non-parametric conditions.

## Results

As shown in the study diagram (Figure 1), 56 women were eligible. Participants were randomly allocated to the intervention and control groups. During the study, 5 women from intervention [pregnancy (*n* = 1), discontinued intervention (*n* = 3), and migration (*n* = 1)] and 4 patients from the control group [discontinued intervention (*n* = 4)] were excluded. Finally, 47 participants completed the study and were consisted in the final analysis. The baseline characteristics of the participants in the present analysis were shown in Table 1. No significant changes were found between groups in demographic characteristics, age, BMI, and physical activity.

**Table 1:** General characteristics of study participants.

Variables	Intervention	Control	*p-*value
	*n* = 23	*n* = 24	
Age [Mean (SD)]	35.09 (8.38)	36.08 (8.58)	0.689^1^
Marital [*n* (%)]			0.529^2^
Single	4 (17.4)	5 (20.8)	
Married	19 (82.6)	19 (79.2)	
Child [*n* (%)]			0.462^2^
0	5 (21.7)	6 (26.1)	
1	6 (26.1)	2 (8.7)	
2	10 (43.5)	11 (47.8)	
3	2 (8.7)	4 (17.4)	
Job [*n* (%)]			0.995^2^
Student	2 (8.7)	2 (8.3)	
Employee	5 (21.7)	5 (20.8)	
Housewife	16 (69.6)	17 (70.8)	
Economic [*n* (%)]			0.712^2^
Poor	2 (8.7)	1 (4.2)	
Average	14 (60.9)	17 (70.8)	
Good	7 (30.4)	6 (25)	
Education [*n* (%)]			0.924^2^
Below diploma	4 (17.4)	4 (16.7)	
Diploma	12 (52.2)	11 (45.8)	
Bachelor and above	7 (30.4)	9 (37.5)	
Physical activity	26.91 (8.60)	28.83 (6.24)	0.385^1^
[Mean (SD)]			
(METs.hr/day)			
BMI [Mean (SD)]	31.63 (3.11)	31.58 (3.66)	0.617^3^
(kg/m^2^)			

^1^*p*-value: Independent samples *t*-test. ^2^*p*-value: Fisher’s Exact test. ^3^*p*-value: Mann-Whitney *U*-test.

### Primary outcomes

After an 8-week follow-up, a higher, significant, decrease in body weight (kg) [−5.23 (1.73) vs. −3.15 (0.88); *P* <0.001] and BMI (kg/m^2^) [−2.05 (0.66) vs. −1.17 (0.34); *P*< 0.001] was observed in ADMF group in comparison to CR. The significant differences weren’t observed in the change of PSQI [−0.39 (1.43) vs. −0.45 (1.88); *P*= 0.73] and ESS [−0.22 (1.24) vs. −0.54 (1.67); *P*= 0.43] between 2 groups. Also, no significant differences were found in physical activity before and after the intervention, in the ADMF group [26.65 (8.19) vs. 26.91 (8.60); *P* = 0.309] and CR group [28.67 (6.17) vs. 28.83 (6.24); *P* = 0.553] (Table 2). The adverse effects related to following the diets weren’t reported among the intervention or control group all over the study.

**Table 2:** Weight, BMI, sleep indexes, and physical activity at baseline and after the 8-week.

Characteristics	Group	Baseline	After 8 weeks	Change	*p*-value^1^	*p*-value^2^
Weight (kg)	ADMF	80.31 (12.64)	75.07 (12.26)	−5.23 (1.73)	<0.001	<0.001
	CR	82.17 (13.43)	79.02 (12.96)	−3.15 (0.88)	<0.001	
BMI (kg/m^2^)	ADMF	31.63 (3.11)	29.58 (3.17)	−2.05 (0.66)	<0.001	<0.001
	CR	31.58 (3.66)	30.42 (3.58)	−1.17 (0.34)	<0.001	
PSQI (score)	ADMF	4.08 (1.50)	3.69 (1.32)	−0.39 (1.43)	0.247	0.735
	CR	3.45 (2.22)	3.00 (1.21)	−0.45 (1.88)	0.220	
ESS (score)	ADMF	5.65 (2.80)	5.43 (2.84)	−0.22 (1.24)	0.393	0.431
	CR	7.08 (2.91)	6.54 (2.58)	−0.54 (1.67)	0.155	
Physical activity (METs.hr/day)	ADMF	26.91 (8.60)	26.65 (8.19)	0.26 (1.21)	0.309^3^	0.775^4^
	CR	28.83 (6.24)	28.67 (6.17)	0.15 (1.28)	0.553^3^	

Values reported as Mean (SD). ^1^*p*-value: Wilcoxon. ^2^*p*-value: Mann- Whitney *U*-test. ^3^*p*-value: Paired *t*-test. ^4^*p*-value: Independent samples *t*-test. ADMF, Alternate-day Modified Fasting; CR, Daily Calorie Restriction; BMI, Body mass index; PSQI, Pittsburgh Sleep Quality Index; ESS, Epworth sleepiness scale.

### Secondary outcomes

Following the ADMF diet led to significant positive changes in SSQ [−0.69 (0.47) vs. −0.08 (0.40); *P*= <0.001] and daytime dysfunction [−0.65 (0.57) vs. 0.04 (0.75); *P*: 0.001] in compare with CR diet. However, significant negative changes were seen in sleep latency [0.87 (0.69) vs. −0.17 (1.01); *P*= <0.001] and sleep duration [0.52 (0.66) vs. −0.12 (0.68); *P*= 0.002] and in parameters of habitual sleep efficiency [0.04 (0.63) vs. 0.00 (0.72); *P*= 0.83] and sleep disturbances [−0.13 (0.75) vs. −0.21 (0.77); *P*= 0.71] no significant changes were observed between groups (Table 3).

**Table 3:** Effects of ADMF and CR on PSQI parameters.

Characteristics	Group	Baseline (score*)	After 8 weeks (score)	Change	*p*-value**	*p*-value***
SSQ	ADMF	0.96 (0.63)	0.26 (0.44)	−0.69 (0.47)	<0.001	<0.001
	CR	0.46 (0.58)	0.37 (0.57)	−0.08 (0.40)	0.317	
Sleep latency	ADMF	0.35 (0.57)	1.22 (0.67)	0.87 (0.69)	<0.001	<0.001
	CR	0.75 (0.84)	0.58 (0.50)	−0.17 (1.01)	0.475	
Sleep duration	ADMF	0.26 (0.61)	0.78 (0.73)	0.52 (0.66)	0.003	0.002
	CR	0.63 (0.71)	0.50 (0.59)	−0.12 (0.68)	0.366	
Habitual sleep efficiency	ADMF	0.35 (0.57)	0.39 (0.49)	0.04 (0.63)	0.739	0.831
	CR	0.33 (0.48)	0.33 (0.48)	0.00 (0.72)	0.900	
Sleep disturbances	ADMF	0.78 (0.79)	0.65 (0.64)	−0.13 (0.75)	0.405	0.706
	CR	0.63 (0.71)	0.42 (0.50)	−0.21 (0.77)	0.197	
Daytime dysfunction	ADMF	0.96 (0.56)	0.30 (0.47)	−0.65 (0.57)	<0.001	0.001
	CR	0.42 (0.58)	0.46 (0.58)	0.04 (0.75)	0.782	

*Values reported as Mean (SD). **p-value: Wilcoxon. ***p-value: Mann-Whitney *U-*test. ADMF, Alternate-day Modified Fasting; CR, Daily Calorie Restriction; SSQ, Subjective sleep quality.

## Discussion

The present study indicated that following the ADMF diet for 8 weeks among women with obesity or overweight improved BMI, body weight, SSQ, and daytime dysfunction compared to CR, however, had no effect on PSQI, ESS, habitual sleep efficiency, and sleep disturbances and harmed sleep duration and sleep latency. Our research indicated that although both diets (ADMF and CR) could result in weight loss after 8 weeks; the effect of the ADMF diet on body weight and BMI loss was higher than the CR. This finding was consistent with several previous studies, for example, Johnson et al. (25) and Razavi et al. (26). In contrast, the study by Trepanowski et al., which lasted for 6 months, did not show any significant beneficial effect on weight loss. While short-term fasting may promote greater weight loss than traditional diets, this effect may not be significant in longer interventions. In fact, other studies have suggested that longer intermittent fasting diets can lead to greater weight loss, and researchers have proposed that intermittent fasting may be a useful dietary method for obese individuals (27, 28). However, the reasons for the discrepancies in anthropometric indices observed in these studies are not well understood, and more long- and short-term trials are needed to fully evaluate the effectiveness of the intermittent fasting. ADMF has exhibited greater participant compliance compared to daily calorie restriction, for longer periods (29). In conventional CR diets, calorie intake must be restricted every day (30), however, the ADMF diet requires food restriction every other day which increases compliance with the diet (29). A decrease in body weight is directly associated with the degree of adherence to the diet (27) and the high adherence rate to the ADMF diet leads to significant weight loss. It has been reported that fasting-induced weight loss is mainly from body fat tissue reduction, while muscle mass is usually preserved during a fasting diet (31). As subjects in ADMF require to fast 3–4 days a week, more decrease in weight is often observed in such diets in comparison with CR (32). The energy balance manages body weight changes (33). During fasting hours, less glucose is available to the body, hence, fat and ketones are considered the main source of energy, and therefore, a decrease in weight and fat tissue will occur (34, 35). The result of several studies indicated that participants in the fasting group reported lower appetite after the intervention. The changes in appetite-regulating hormones may have changed people’s appetite. In animal trials, treatment with alternate-day fasting increased adiponectin concentrations, while decreasing leptin and resistin (36).

Sleep disturbances are common findings in obese people (37), which affects not only people with extreme obesity but also people with medium obesity. Excessive fat tissue especially visceral adipose plays a main role in this relation (38). Indeed, visceral adipose tissue increases the secretion of inflammatory cytokines (TNF-α, IL-1, and IL-6). These cytokines cause low-grade chronic inflammation (39). The studies reported that some pro-inflammatory cytokines could have a role in sleep regulation (40). Especially, IL-1β and TNF-α have circadian secretion, with the maximum TNF-α and IL-6 secretion between 01:00 to 02:00 a.m., thus they can regulate the physiology of sleep in both humans and animals (41). In individuals with obesity, IL-6 and TNF-α have more secretion in the morning instead of the night and are related to BMI and sleep disorder (42). Therefore, these findings propose a hypothetical vicious circle including pro-inflammatory cytokines, obesity, and sleep disorder (3). Although our intervention did not affect the total PSQI score and ESS score, subscales of PSQI such as daytime dysfunction and SSQ improved significantly in the ADMF group. While the subscales of sleep latency and duration significantly worsened in the intervention group. It can be said that ADMF has increased sleep quality and daily function. However, the findings of various studies are inconsistent and further research is needed to better understand the possibility of a relationship between adherence to the ADMF diet and sleep quality. There are some limitations in our study. The sample size and duration of the study were relatively short. Nevertheless, in this short follow-up, we showed the effects of ADMF on body weight and some sleep indices. Another limitation of our study is that the assessment of sleep quality and daytime sleepiness was based only on self-reported questionnaires, which might have resulted in misstatements. We determined compliance with the prescribed diet using a food record questionnaire. Forms were completed by the patients three days a week, once every two weeks. Also, we monitored individuals via phone interviews all over the study.

## Conclusion

Since obesity has been suggested as one of the main causes of sleep disorders, weight loss diets may play a role in sleep quality. The present study suggests that ADMF is an efficacious dietary method for decreasing body weight and managing BMI in women with obesity or overweight. In addition, we indicated that an ADMF diet can increase SSQ and improve daytime dysfunction, in comparison with a CR diet. These results give us better insight into the ability of ADMF versus CR for the management of weight and BMI. However, more studies are required to address the direction of causality and generalize the results to other diverse population groups with different health statuses. Moreover, more research is required to study the long-term effects of ADMF on holistic and metabolic health. The findings of this study will increase our information on fasting diets, which can be applied to ameliorate dietary recommendations. The addressing of the public health effect of sleep behaviors in the prevention of chronic diseases corroborates this call for further research.

## Data availability statement

The raw data supporting the conclusions of this article will be made available by the authors, without undue reservation.

## Ethics statement

The studies involving human participants were reviewed and approved by the Ethics Committee of Kashan University of Medical Sciences (IR.KAUMS.MEDNT.REC.1401.046). The patients/participants provided their written informed consent to participate in this study.

## Author contributions

SJ conceived the trial, designed the experiment, and was the Chief Investigator. AY analyzed the data and was responsible for the statistical design of the study. SH assisted with the conduction of the study and wrote the manuscript. All study authors read and approved the final version of the manuscript.

## Funding

This work was financially supported by the Kashan University of Medical Sciences and Health Services (Kashan, Iran).

## Conflict of interest

The authors declare that the research was conducted in the absence of any commercial or financial relationships that could be construed as a potential conflict of interest.

## Publisher’s note

All claims expressed in this article are solely those of the authors and do not necessarily represent those of their affiliated organizations, or those of the publisher, the editors and the reviewers. Any product that may be evaluated in this article, or claim that may be made by its manufacturer, is not guaranteed or endorsed by the publisher.

## Abbreviations

BMI: Body mass index
ADMF: Alternate-day Modified Fasting
CR: Daily calorie restriction
ESS: Epworth sleepiness scale
PSQI: Pittsburgh sleep quality index
SSQ: Subjective sleep quality
MET: Metabolic equivalents
